# Core column prediction for protein multiple sequence alignments

**DOI:** 10.1186/s13015-017-0102-3

**Published:** 2017-04-19

**Authors:** Dan DeBlasio, John Kececioglu

**Affiliations:** 1 Department of Computer Science, The University of Arizona, Tucson, AZ 85721 USA; 2 Computational Biology Department, Carnegie Mellon University, Pittsburgh, PA 15213 USA

**Keywords:** Multiple sequence alignment, Core blocks, Alignment accuracy, Accuracy estimation, Parameter advising, Machine learning, Regression

## Abstract

**Background:**

In a computed protein multiple sequence alignment, the *coreness* of a column is the fraction of its substitutions that are in so-called core columns of the gold-standard reference alignment of its proteins. In benchmark suites of protein reference alignments, the core columns of the reference alignment are those that can be confidently labeled as correct, usually due to all residues in the column being sufficiently close in the spatial superposition of the known three-dimensional structures of the proteins. Typically the accuracy of a protein multiple sequence alignment that has been computed for a benchmark is only measured with respect to the core columns of the reference alignment. When computing an alignment in practice, however, a reference alignment is not known, so the coreness of its columns can only be predicted.

**Results:**

We develop for the first time a *predictor* of column coreness for protein multiple sequence alignments. This allows us to predict which columns of a computed alignment are core, and hence better estimate the alignment’s accuracy. Our approach to predicting coreness is similar to nearest-neighbor classification from machine learning, except we transform nearest-neighbor distances into a coreness prediction via a regression function, and we learn an appropriate distance function through a new optimization formulation that solves a large-scale linear programming problem. We apply our coreness predictor to *parameter advising*, the task of choosing parameter values for an aligner’s scoring function to obtain a more accurate alignment of a specific set of sequences. We show that for this task, our predictor strongly outperforms other column-confidence estimators from the literature, and affords a substantial boost in alignment accuracy.

## Background

The accuracy of a multiple sequence alignment computed on a benchmark set of input sequences is usually measured with respect to a *reference alignment* that represents the gold-standard alignment of the sequences. For protein sequences, reference alignments are often determined by structural superposition of the known three-dimensional structures of the proteins in the benchmark. The *accuracy* of a computed alignment is then defined to be the fraction of pairs of residues aligned in the so-called core columns of the reference alignment that are also present in columns of the computed alignment. *Core columns* represent those in the reference that are deemed to be reliable, which can be objectively defined as those columns containing a residue from every input sequence such that the pairwise distances between these residues in the structural superposition of the proteins are all within some threshold (typically a few angstroms). In short, given a known reference alignment whose columns are labeled as either core or non-core, we can determine the accuracy of any other computed alignment of its proteins by evaluating the fraction of aligned residue pairs from these core columns that are recovered.

For a column in a computed alignment, we can also define the *coreness* value for the column to be the fraction of its aligned residue pairs that are in core columns of the reference alignment. (Note this definition of column coreness is fully objective when core columns are identified through automated superposition of known protein structures, as done for example in the PALI [1] benchmark suite.) A coreness value of 1 means the column of the computed alignment corresponds to a core column of the reference alignment.

When aligning sequences in practice, obviously such a reference alignment is not known, and the accuracy of a computed alignment, or the coreness of its columns, can only be estimated. A good *accuracy estimator* for computed alignments is extremely useful [2]. It can be leveraged to

- pick among alternate alignments of the same sequences the one of highest estimated accuracy, for example, to choose good parameter values for an aligner’s scoring function as in *parameter advising* [3, 4]; or

- select the best result from an ensemble of different aligners, naturally yielding a new *ensemble aligner*, which can be far more accurate than any of its individual aligners  [5].

Similarly, a good *coreness predictor* for columns in a computed alignment can be used to

- mask out unreliable regions of the alignment before computing an evolutionary tree, to boost the quality of phylogeny reconstruction; or

- improve an alignment accuracy estimator by concentrating its evaluation function on columns of higher predicted coreness, thereby boosting the performance of parameter advising.

In fact, a perfect coreness predictor by itself would in principle yield an ideal accuracy estimator.

In this paper, we develop for the first time a column coreness predictor for protein multiple sequence alignments. Our approach to predicting coreness is similar in some respects to nearest-neighbor classification from machine learning, except we transform nearest-neighbor distance into a coreness prediction via a regression function, and we learn an appropriate distance function through a new optimization formulation that solves a large-scale linear programming problem. We leverage our new coreness predictor to yield an improved alignment accuracy estimator, and evaluate its performance by applying the improved estimator to the task of parameter advising in multiple sequence alignment.

### Related work

To our knowledge, this is the first fully general attempt to directly predict the coreness of columns in computed protein alignments. Tools are available that assess the quality of columns in a multiple alignment, and can be categorized into: (a) those that only identify columns as unreliable, for removal from further analysis; and (b) those that compute a column quality score, which can be thresholded. Tools that simply mask unreliable columns of an alignment include GBLOCKS [6], TrimAL [7], and ALISCORE [8]. Popular quality-score tools are Noisy [9], ZORRO [10], TCS [11], and GUIDANCE [12].

Our experiments compare our coreness predictor to TCS and ZORRO: the most recent tools that provide quality scores, as opposed to masking columns. Among the other quality-score tools listed above, Noisy has been shown to be dominated by GUIDANCE, which is in turn dominated by ZORRO. (GUIDANCE also requires four or more sequences, which excludes many benchmarks.) Below we briefly summarize the approaches behind TCS and ZORRO.

TCS (short for “transitive consistency score”) extends an earlier approach of COFFEE [13]. For a pair *i*, *j* of residues that are aligned in a column and that come from sequences *A* and *B*, the *support* for aligned pair *i*, *j* is the sum of the scores of all pairwise alignments of every other sequence *C* versus *A* and *B*, where the pairwise alignments involving *C* are constrained to align *i* and *j* to a common residue of *C*, and where this sum is normalized so support is in the range [0, 1]. The TCS score for a column is then the average support of its aligned residue pairs.

ZORRO uses an evolutionary tree over the alignment’s sequences to determine a weight for each sequence pair. The length of each edge in the tree is apportioned among the sequence pairs whose tree paths include that edge; the total amount of edge length apportioned to a given sequence pair yields a *weight* for that pair, where these weights also take into account both an estimate of the evolutionary distance between sequences (estimated by the length of the tree path between them), and the correlation between sequence pairs (estimated by the length of overlap in the paths between the pairs). The ZORRO score for a column is then the weighted sum, over the column’s aligned residue pairs, of the probability of emitting the residue pair’s amino acids by a pair hidden Markov model, times the weight of the residue pair’s corresponding sequence pair.

In contrast to the quality scores of TCS and ZORRO, we directly predict column coreness. Our approach is also not dependent on the choice of an alignment scoring scheme as in TCS, or the choice of hidden Markov model emission probabilities as in ZORRO.

### Plan of the paper

"Learning a coreness predictor" section describes how we learn our coreness predictor. We then explain how we use predicted coreness to improve accuracy estimation for protein alignments. "Assessing the coreness predictor" section evaluates our approach to coreness prediction by applying the improved accuracy estimator to alignment parameter advising. Finally, we conclude and give directions for further research.

## Learning a coreness predictor

To describe how we learn a column coreness predictor, we first discuss our *representation* of alignment columns, and our grouping of consecutive columns into *window classes*. We then present our *regression function* for predicting coreness, which transforms the nearest-neighbor distance from a window to a class into a coreness value. Following this we explain how to learn the window distance function by solving a large-scale *linear programming* problem. Finally we show that the resulting window distances satisfy the *triangle inequality*, which enables the use of data structures for metric-space nearest-neighbor search when evaluating the regression function.

### Representing alignment columns

The information used by our coreness predictor, beyond the multiple sequence alignment itself, is an annotation of its protein sequences by predicted secondary structure (which can be obtained in a preprocessing step by running the sequences through a standard protein secondary structure prediction tool such as PSIPRED [14]). When inputting a column from such an annotated alignment to our coreness predictor, we need a column representation that, while capturing the association of amino acids and predicted secondary structure types, is also independent of the number of sequences in the column. This is necessary as our predictor will be trained on example alignments of particular sizes, yet the resulting predictor must apply to alignments with arbitrary numbers of sequences.

Let $\Sigma$ be the 20-letter amino acid alphabet, and $\Gamma = \{\alpha , \beta , \gamma \}$ be the secondary structure alphabet, corresponding respectively to types $\alpha$ *-helix*, $\beta$ *-strand*, and *other* (also called *coil*). We encode the association of an amino acid $c \in \Sigma$ with its predicted secondary structure type $s \in \Gamma$ by an ordered pair (*c*, *s*) that we call a *state*, from the set  $Q = (\Sigma \times \Gamma ) \,\cup \, \{\xi \}$. Here $\xi = (\texttt {-},\texttt {-})$ is the *gap state*, where the dash symbol $\hbox{`--'}\not \in \Sigma$ is the alignment *gap character*.

We represent a multiple alignment column as a distribution over the set of states *Q*, which we call its *profile* (mirroring standard terminology [15, p. 101]). We denote the profile *C* for a given column by a function *C*(*q*) on states ${q \in Q}$ satisfying $C(q) \ge 0$ and $\sum _{q \in Q} C(q) = 1$. Most secondary structure prediction tools output a confidence value (not a true probability) that an amino acid in a protein sequence has a given secondary structure type. For a column of amino acids $(c_1 \cdots c_k)$ in a multiple alignment of *k* sequences, denote the *confidence* that amino acid $c_i$ has secondary structure type $s \in \Gamma$ by $p_i(s) \ge 0$, where $\sum _{s \in \Gamma } \, p_i(s) = 1$. For non-gap state ${q = (a,s) \ne \xi }$, profile *C* has value

$$\begin{aligned} C(q)\,:=\, \frac{1}{k} \, \sum _{1 \le i \le k\,:\, c_i = a} p_i(s). \end{aligned}$$

In other words, *C*(*q*) is the normalized total confidence across the column in state $q \ne \xi$. For gap state $q = \xi$, the profile value is

$$\begin{aligned} C(\xi )\,:=\, \frac{1}{k}\, \Bigl | \bigl \{ i\,: \, c_i = \hbox{`--'} \bigr \} \Bigr |, \end{aligned}$$

the relative frequency of gap characters in the column.

### Classes of column windows

In protein benchmarks, a column of a reference alignment is labeled core if the residues in that column are all sufficiently close in the structural superposition of the known three-dimensional structures of the proteins. The folded structure around a residue is not simply a function of the amino acid of the residue itself, or its secondary structure type, but is also a function of nearby residues in the protein. Consequently, to predict the coreness of a column in a computed alignment, we need contextual information from nearby columns of the alignment. We gather this additional context around a column by forming a window of consecutive columns centered on the given column. Formally, a *window* *W* of width ${w \ge 1}$ is a sequence of $2w \!+\! 1$ consecutive column profiles $C_{-w} \cdots C_{-1} C_0 C_{+1} \cdots C_{+w}$ centered around profile $C_0$.

We define the following set of *window classes* $\mathcal C$, depending on whether the columns in a labeled training window are known to be core or non-core in the reference alignment. (When later extracting training windows from a computed alignment that has a known reference alignment, we will label a column in a computed alignment as core iff its true coreness value—namely, the fraction of its residue pairs that are in core columns of the reference alignment—is above a fixed threshold.) We denote a column labeled core by C, and a column labeled non-core by N. For window width $w \!=\! 1$ (which has three consecutive columns), such labeled windows correspond to strings of length 3 over alphabet $\{\texttt {C}, \texttt {N}\}$. The three classes of *core windows* are CCC, CCN, NCC; the three classes of *non-core windows* are CNN, NNC, NNN. (A window is considered core or non-core depending on the label of its center column. We exclude windows NCN and CNC, as these almost never occur in reference alignments.) Together these six classes comprise set $\mathcal C$. We call the five classes with at least one core column C in the window, *structured classes*; the one class with no core columns is the *unstructured class*, denoted by $\bot = \texttt {NNN}$.

### The coreness regression function

We learn a coreness predictor by fitting a regression function that first measures the similarity between a column’s window and training examples of windows with known coreness, and then transforms this similarity into a coreness value.

The similarity of windows ${V = V_{-w} \cdots V_w}$ and ${W = W_{-w} \cdots W_w}$ is expressed in terms of the similarity of their corresponding column profiles $V_i$ and $W_i$. We measure the dissimilarity of two such profiles from window class *c* at position *i*, using class- and position-specific *substitution scores* $\sigma _{c,i}(p,q)$ on pairs of states *p*, *q*. (We describe in later sections how we learn these scores.) Given substitution scores $\sigma _{c,i}$, the *distance* between windows *V* and *W* from structured class ${c \in {\mathcal C}-\{\bot \}}$ is

$$\begin{aligned} d_c(V,W)\,:= \sum _{-w \,\le i \,\le \, +w} \, \sum _{p,q \,\in \, Q} \, V_i(p)\, W_i(q)\,\, \sigma _{c,i}(p,q). \end{aligned}$$

These positional $\sigma _{c,i}$ allow distance function $d_c$ to score dissimilarity higher at positions *i* near the center of the window, and lower towards its edges. These class-specific $\sigma _{c,i}$ also allow distance functions to score dissimilarity differently for core and non-core classes.

The *regression function* that predicts the coreness of a column first forms a window *W* centered on the column, and then performs the following.

1. (*Find distance to closest class*) Across all labeled training windows, in all structured window classes, find the training window that has smallest class-specific distance to *W*. Call this closest window *V*, its class *c*, and their distance $\delta = d_c(V,W)$.
2. (*Transform distance to coreness*) If class *c* is a core class, return the coreness value given by transform function $f_\text {core}(\delta )$. Otherwise, return value $f_\text {non}(\delta )$.

Note this uses two different transform functions to map distance to coreness: function $f_\text {core}$ for core classes, and $f_\text {non}$ for non-core.

We next explain how we efficiently find distance $\delta$, and then describe the transform functions *f*.

#### Finding the distance to a class

To find the distance of a window *W* to a class *c*, we need to find the *nearest neighbor* of *W* among the set of training windows $T_c$ in class *c*, namely $\mathop {\mathrm {argmin}}_{V \in T_c} \bigl \{ d_c(V,W) \bigr \}$. Finding the nearest neighbor through exhaustive search by explicitly evaluating $d_c(V,W)$ for every window *V* can be expensive when $T_c$ is large (and cannot be avoided in the absence of exploitable properties of function $d_c$).

When the distance function is a *metric*, for which the key property is the *triangle inequality* (namely that $d(x,z) \,\le \, d(x,y) + d(y,z)$ for any three objects *x*, *y*, *z*), faster nearest neighbor search is possible. In this situation, in a preprocessing step we can first build a data structure over the set $T_c$, which then allows us to perform faster nearest neighbor searches on $T_c$ for any query window *W*. One of the best data structures for nearest neighbor search under a metric is the *cover tree* of Beygelzimer, Kakade and Langford [16]. Theoretically, cover trees permit nearest neighbor searches over a set of *n* objects in $O(\log n)$ time, after constructing a cover tree in $O(n \log n)$ time, assuming that the intrinsic dimension of the set under metric *d* has a so-called bounded expansion constant [16]. (For actual data, the expansion constant can be exponential in the intrinsic dimension.) In our experiments, for nearest neighbor search we use the recently-developed *dispersion tree* data structure of Woerner and Kececioglu [17], which in extensive testing on scientific data is significantly faster in practice than cover trees.

We build a separate dispersion tree for each structured window class $c \in {\mathcal C} - \{\bot \}$ over its training set $T_c$ using its distance function $d_c$ in a preprocessing step. To find the nearest neighbor to window *W* over all training windows  ${\mathcal T} = \bigcup _{c} T_c$ we then perform a nearest neighbor search with *W* on the dispersion tree for each structured class *c*, and merge these $|{\mathcal C}|-1$ search results by picking the one with smallest distance to *W*.

#### Transforming distance to coreness

**Fig. 1:**
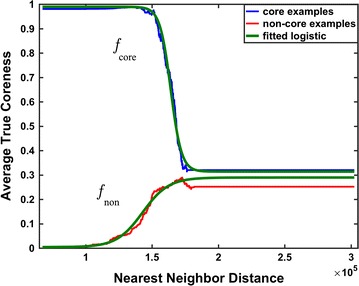
Fit of the distance transform functions to true coreness. The *blue* and *red* curves track on the vertical axis the average true coreness of training examples, whose nearest neighbor is respectively from a core or non-core class, at their corresponding nearest-neighbor distance on the horizontal axis. Average coreness is computed as described earlier with count $\ell = 100$. The *top* and *bottom green* curves respectively show the fitted logistic functions $f_\text {core}$ and $f_\text {non}$ that are used by the coreness regressor to transform nearest-neighbor distance into predicted coreness

To transform the *nearest-neighbor distance* $\delta$ from Step (1) of the regression procedure into a coreness value in Step (2), we use logistic functions for $f_\text {core}$ and $f_\text {non}$. We fit these logistic functions to empirically-measured average-coreness values at nearest-neighbor distances collected for either core or non-core training examples, using the curve-fitting tools in SciPy [18]. The form of the *logistic function* we use is

$$\begin{aligned} f(x)\,:=\, \kappa \,+ \,(\lambda -\kappa ) \, \frac{1}{1+ e^{-\alpha (x\,-\,\beta )}}, \end{aligned}$$

where parameters $\kappa$ and $\lambda$ are respectively the minimum and maximum average-coreness values measured on the examples, while parameters $\alpha$ and $\beta$ respectively control the slope and location of the logistic function’s inflection point. For function $f_\text {core}$ parameter $\alpha$ is positive (so coreness decreases with distance to a *core* class); for $f_\text {non}$parameter $\alpha$ is negative (so coreness increases with distance from a *non-core* class). As Fig. 1 later shows, these logistic transform functions fit actual coreness data remarkably well.

For the fitting process, we first learn the distance functions $d_c$ as described in "Learning the distance function by linear programming" section, and then fit the transform functions to empirical coreness values measured at the distances observed for example windows from our set of training windows. To fit function $f_\text {core}$ we

- take the examples whose nearest neighbor is from one of the three *core* classes,
- sort these examples by their observed nearest-neighbor distance,
- at each observed distance $\delta$, collect all $k \!\ge \! 1$ examples whose distance equals $\delta$, the $\ell$ successive examples whose distance is below $\delta$, and the $\ell$ successive examples above $\delta$, where count $\ell$ is fixed for the fitting process, and
- compute the average true-coreness value of these $k + 2\ell$ examples, and associate this average value with distance $\delta$.

A logistic curve is then fit to these pairs of average true-coreness and observed nearest-neighbor distances. To fit function $f_\text {non}$, this same process is repeated separately for examples whose nearest neighbor is from one of the two structured *non-core* classes.

To predict coreness for a window from a computed alignment, again we (1) find its nearest-neighbor distance $\delta$ among all training windows from structured classes, and (2) transform this distance to coreness by returning $f_\text {core}(\delta )$ if the nearest neighbor is from a core class and $f_\text {non}(\delta )$ otherwise.

### Learning the distance function by linear programming

We now describe the linear program used to learn the distance functions on column windows. Again we divide the window classes $\mathcal C$ into two categories: the *structured classes*, containing windows centered on core columns, or centered on non-core columns that are flanked on at least one side by core columns; and the *unstructured class*, containing windows of only non-core columns. We again denote this unstructured class of completely non-core windows by $\bot \in {\mathcal C}$. The linear program learns a *class-specific* distance function $d_c$ for each structured window class  $c \,\in \, {\mathcal C} - \{\bot \}$.

In principle, the linear program tries to find distance functions $d_c$ that make the following “conceptual” nearest-neighbor classifier accurate. (We do not actually learn such a classifier, but instead ultimately learn a regressor.) This classifier forms a window *W* centered on the column to be classified, and finds the nearest neighbor to *W* over all *structured* classes ${\mathcal C} - \{\bot \}$ in the training set, using their corresponding distance functions $d_c$. Let the distance to this nearest neighbor be $\delta$, and its structured class be *c*. The conceptual classifier would then compare distance $\delta$ to a threshold $\tau$.

- If $\delta \,\le \, \tau$, the central column of window *W* is declared to be “core” or “non-core” depending on whether structured class *c* is respectively core or non-core.
- Otherwise, window *W* is deemed to be in the unstructured non-core class $\bot$, and its central column is declared “non-core.”

The key aspect of this conceptual nearest-neighbor classifier is that it can recognize a *completely non-core* window *W* from class $\bot$, without actually having any examples in its training set that are close to *W*. This is crucial, as the set of possible windows from the unstructured class $\bot$ is enormous and may lack any recognizable structure, which would make reliably identifying windows from class $\bot$ by having a near neighbor in the training set hopeless. On the other hand, identifying windows from the structured classes is possible by having enough examples in the training set. The following linear program learns both distance functions $d_c$ and distance threshold $\tau$.

To construct the linear program, we partition the *training set* $\mathcal T$ of labeled windows by window class: subset $T_c \subseteq {\mathcal T}$ contains all training windows of class $c \in {\mathcal C}$. We then form a smaller *training sample* ${S_c \subseteq T_c}$ for each class *c* by choosing a random subset of $T_c$ with a specified cardinality $|S_c|$.

For a sample training window $W \in S_c$ we identify other windows $V \in T_c$ from the same class *c* in the full training set that are close to *W* (under a default distance $\widetilde{d}_c$). We call these close windows *V* from the same class *c*, *targets*. Similarly for $W \in S_c$ we identify other windows $U \in T_b$ from a different class $b \ne c$ in the full training set that are also close to *W* (under $\widetilde{d}_b$). We call these other close windows *U* from a different class *b*, *impostors* (paralleling the terminology of Weinberger and Saul [19]).

We call these sets of windows that are close to a given window *W* the *neighborhood* ${\mathcal N}_c(W,i)$ of *W* for a structured class $c \,\in \, {\mathcal C} - \{\bot \}$, which denotes the set of *i*-nearest-neighbors to *W* (not including *W*) from training set $T_c$ under the class-specific *default distance* function $\widetilde{d}_c$. (The default distance function that we use in our experiments is described later.)

At a high level, the linear program finds a distance function that, for sample windows ${W \in S_c}$

- pulls in targets $V \,\in \, {\mathcal N}_c(W,i)$, by making $d_c(V,W)$ small, and
- pushes away impostors ${U \,\in \, {\mathcal N}_b(W,i)}$ for $b \ne c$, by making $d_b(U,W)$ large.

The neighborhoods ${\mathcal N}(W,i)$ that give these sets of targets and impostors are defined with respect to *default* distance functions $\widetilde{d}$. Ideally these neighborhoods should be defined with respect to the *learned* distance functions $d_c$, but obviously these learned distances are not available until after the linear program is solved. We address this discrepancy by *iteratively* solving a series of linear programs. The first linear program at iteration 1 defines neighborhoods with respect to distance functions $d^{(0)} = \widetilde{d}$, and its solution yields the new functions $d^{(1)}$. In general, iteration *i* uses the previous iteration’s functions $d^{(i-1)}$ to formulate a linear program whose solution yields the new distance functions $d^{(i)}$. This process is repeated for a fixed number of iterations, or until the change in the distance functions is sufficiently small.

The *target constraints* of the linear program, for each sample window $W \in S_c$ from each structured class $c \,\in \, {\mathcal C} - \{\bot \}$, and each target window $V \,\in \, {\mathcal N}_c(W,k)$, are

1 $$\begin{aligned} e_{VW}\,\ge\, & \, d_c(V,W) - \tau, \end{aligned}$$

2 $$\begin{aligned} e_{VW}\,\ge\, & \, 0,\end{aligned}$$

where $e_{VW}$ is a target *error variable* and $\tau$ is a *threshold variable*. In the above, quantity $d_c(V,W)$ is a linear expression in the *substitution score variables* $\sigma _{c,i}(p,q)$, so constraint (1) is a linear inequality in all these variables. Intuitively, we would like condition $d_c(V,W) \,\le \, \tau$ to hold (so *W* will be considered to be in its correct class *c*); in the solution to the linear program, variable $e_{VW}$ will equal $\max \bigl \{d_c(V,W) - \tau , \, 0\bigr \}$, the amount of error by which this ideal condition is violated.

In the target neighborhood ${\mathcal N}_c(W,k)$ above, count *k* specifies the number of targets for each sample window *W*. In our experiments we use a small number of targets, with $k = 2$ or 3.

The *impostor constraints* for each sample window $W \in S_c$ from each structured class $c \,\in \, {\mathcal C} - \{\bot \}$, and each impostor window $V \,\in \, {\mathcal N}_b(W,\ell )$ from each structured class $b \,\in \, {\mathcal C} - \{\bot \}$ with $b \ne c$, are

3 $$\begin{aligned} f_W\,\ge\, & \, \tau - d_b(V,W) + 1,\end{aligned}$$

4 $$\begin{aligned} f_W\,\ge\, & \, 0,\end{aligned}$$

where $f_W$ is an impostor error variable. Intuitively, we would like condition ${d_b(V,W) \,>\, \tau }$ to hold (so *W* will not be considered to be in the incorrect class *b*), which we can express by ${d_b(V,W) \,\ge \, \tau + 1}$ using a *margin* of 1. (Since the scale of the distance functions is arbitrary, we can always pick a unit margin without loss of generality.) In the solution to the linear program, variable $f_W$ will equal ${\max _{b \,\in \, {\mathcal C}-\{\bot \}, \,\, V \,\in \, {\mathcal N}_b(W,\,\ell )} \bigl \{\tau - d_b(V,W) + 1, \, 0 \bigr \}}$, the largest amount of error by which this condition is violated for *W* across all *b* and *V*.

We also have impostor constraints for each completely non-core window ${W \in T_\bot }$ and each core window $V \in {\mathcal N}_b(W,\ell )$ from each structured core class *b* (as we do not want *W* to be considered core), which are of the same form as inequalities (3) and (4) above.

In the impostor neighborhood ${\mathcal N}_b(W,\ell )$ above, count $\ell$ specifies the number of impostors for each sample window *W*. We use a large number of impostors $\ell \approx 100$ in our experiments. Having a single impostor error variable $f_W$ per sample window *W* (versus a target error variable $e_{VW}$ for every *W* and target *V*) allows us to use a large count $\ell$ while still keeping the number of variables in the linear program tractable.

The *triangle inequality constraints*, for each structured class ${c \,\in \, {\mathcal C} - \{\bot \}}$, each window position $-w \le i \le w$, and all states $p, q, r \,\in \, Q$ (including the gap state $\xi$), are

5 $$\begin{aligned} \sigma _{c,i}(p,r)\,\le\, & \, \sigma _{c,i}(p,q) + \sigma _{c,i}(q,r). \end{aligned}$$

These reduce to simpler inequalities when states *p*, *q*, *r* are not all distinct or coincide with the gap state (which we do not enumerate here). A consequence of constraint (5) is that the resulting distance functions $d_c$ also satisfy the triangle-inequality property, as we prove in "Ensuring the triangle inequality" section. This property allows us to use faster metric-space data structures for computing the nearest-neighbor distance $\delta$ as discussed earlier.

The remaining constraints, for all structured classes $c \,\in \, {\mathcal C} - \{\bot \}$, positions ${-w \le i \le w}$, states ${p,q \,\in \, Q}$, and gap state $\xi$, are

6 $$\begin{aligned} \sigma _{c,i}(p,q)\,= \,&\, \sigma _{c,i}(q,p),\end{aligned}$$

7 $$\begin{aligned} \sigma _{c,i}(p,p)\,\le\, &\, \sigma _{c,i}(p,q),\end{aligned}$$

8 $$\begin{aligned} \sigma _{c,i}(p,q)\,\ge\, & \, 0,\end{aligned}$$

9 $$\begin{aligned} \sigma _{c,i}(\xi , \xi )\, = \,& \, 0,\end{aligned}$$

10 $$\begin{aligned} \tau\,\ge \, & \, 0,\end{aligned}$$

which ensure the distance functions are symmetric and non-negative. (We do not enforce the other metric conditions $d_c(W,W) = 0$ and $d_c(V,W) > 0$ for $V \ne W$, as these are not needed for our coreness predictor, and we prefer having a less constrained distance $d_c$ that might better minimize the following error objective.)

Finally, the *objective function* minimizes the average error over all training sample windows. Formally, we minimize

$$\begin{aligned} \alpha {\textstyle \frac{1}{|{\mathcal C}| - 1}} \sum _{c \,\in \, {\mathcal C}-\{\bot \}} \, {\textstyle \frac{1}{|S_c|}} \sum _{W \,\in \, S_c} {\textstyle \frac{1}{k}} \sum _{V \,\in \, {\mathcal N}_c(W,k)} e_{VW} + (1-\alpha ) \, {\textstyle \frac{1}{|{\mathcal C}|}} \, \sum _{c \,\in \, {\mathcal C}} \, {\textstyle \frac{1}{|S_c|}} \sum_{W\, \in \,S_c} f_W, \end{aligned}$$

where $0 \le \alpha \le 1$ is a blend parameter controlling the weight on target error versus impostor error. We note that in an optimal solution to this linear program, variables ${e_{VW} = \max \bigl \{ d_c(V,W) - \tau , \, 0 \bigr \}}$ and ${f_W = \max _{V,b} \bigl \{ \tau - d_b(V,W) + 1, \, 0 \bigr \}}$, since inequalities (1)–(4) ensure the error variables are at least these values, while minimizing the above objective function ensures they will not exceed them. Thus solving the linear program finds distance functions $d_c$ given by substitution scores $\sigma _{c,i}(p,q)$ that minimize the average over the training windows $W \in S_c$ of the amount of violation of our ideal conditions $d_c(V,W) \,\le \, \tau$ for targets $V \in T_c$ and $d_b(V,W) > \tau$ for impostors $V \in T_b$.

To summarize, the variables of the linear program are the substitution scores $\sigma _{c,i}(p,q)$, the error variables $e_{VW}$ and $f_W$, and the threshold variable $\tau$. For *n* total training sample windows, *k* targets per sample window, *m* window classes of width *w*, and amino-acid alphabet size *s*, this is $\Theta (k n + s^2 w m)$ total variables. The main constraints are the target constraints, impostor constraints, and triangle inequality constraints. For $\ell$ impostors per sample window, this is ${\Theta \bigl ( (k + \ell m)n + s^3 w m \bigr )}$ total constraints. We ensure that solving the linear program is tractable by controlling the number *k* of targets, the number $\ell$ of impostors, and the total size *n* of the training sample.

### Ensuring the triangle inequality

We now show that the distance functions obtained by solving the above linear program obey the triangle inequality.

#### **Theorem 1**

(Triangle Inequality on Window Distances)* The class distance functions* $d_c$ *obtained by solving the linear program satisfy the triangle inequality.*

#### *Proof*

For every class *c*, and all windows *U*, *V*, and *W*,

11 $$\begin{aligned} d_c(U,W)\,=\, & \sum _i \sum _{p,r}  U_i(p)\, W_i(r)\,  \sigma _{c,i}(p, r)  \\\,=\, &\sum _i \sum _{p,q,r} U_i\,(p) \,V_i (q)\, W_i(r)\, \sigma _{c,i}(p, r) \end{aligned}$$

12 $$\begin{aligned}\,\le\,\, & {} \sum _i \sum _{p,q,r} \, U_i(p) \, V_i(q) \, W_i(r)  \Bigl ( \sigma _{c,i}(p,q) \,+\, \sigma _{c,i}(q,\!r) \Bigr ) \end{aligned}$$

13 $$\begin{aligned}\,=\,\, & \sum _i \sum _{p,q,r}\, U_i(p)\,  V_i(q)\, W_i(r)\,  \sigma _{c,i}(p,q)\\ & +\,\,\sum _i \sum _{p,q,r}\, U_i(p)\, \, V_i(q)\, \, W_i(r)\, \sigma _{c,i}(q, r) \\\,=\,\, & \sum _i \sum _{p,q} \,U_i(p)\, \, V_i(q)\, \, \sigma _{c,i}(p,q) \\ & \quad   +\,\sum _i \sum _{q,r} \,V_i(q)\,\, W_i(r)\, \sigma _{c,i}(q,r) \\\,=\,\, &\, d_c(U,V)\, + \,d_c(V,W),\end{aligned}$$

where equation (11) follows from the identity $\sum _q V_i(q) = 1$, inequality (12) follows from constraint (5) in the linear program, and equation (13) follows from the identities ${\sum _r W_i(r) = \sum _p U_i(p) = 1}$.

In short, ${d_c(U,W) \,\le \, d_c(U,V) + d_c(V,W)}$ for all windows *U*, *V*, *W*, so the triangle inequality holds on distances $d_c$. $\square$

Since window distances satisfy the triangle inequality, we can use fast data structures for metric-space nearest-neighbor search to evaluate the coreness predictor.

## Applying coreness to accuracy estimation

The Facet alignment accuracy estimator [3] is a linear combination of efficiently-computable feature functions of an alignment that are positively correlated with true accuracy. As mentioned earlier, the true accuracy of a computed alignment is measured only with respect to core columns of the reference alignment. We leverage our coreness predictor to improve the Facet estimator by: (1) creating a *new* feature function that attempts to directly estimate true accuracy, and (2) concentrating the evaluation of *existing* feature functions on columns with high predicted coreness.

### Creating a new coreness feature

Our new feature function on alignments, which we call Predicted Alignment Coreness, is similar to the so-called total-column score sometimes used to measure alignment accuracy. Predicted Alignment Coreness counts the number of columns in the alignment that are predicted to be core, by taking a window *W* around each column, and counting the number of windows whose *predicted coreness* value $\chi (W)$ exceeds a threshold $\kappa$. This count of predicted core columns in the given alignment is normalized by an estimate of the number of true core columns in the unknown reference alignment of the sequences.

Formally, the *Predicted Alignment Coreness* feature function $F_\texttt {AC}$ for computed alignment $\mathcal A$ of sequences $\mathcal S$ is

$$\begin{aligned} F_\texttt {AC}(\mathcal {A})\,:=\, \frac{1}{L(\mathcal {S})}\,\Bigl | \bigl \{ W \in \mathcal {A} \, :\,\chi (W) \,\ge \, \kappa \bigr \} \Bigr|, \end{aligned}$$

where the notation $W \in \mathcal {A}$ refers to all windows of columns of $\mathcal A$.

The normalizing function *L* in the denominator is designed to be positively correlated with the number of core columns in the reference alignment for $\mathcal S$. (The normalizer *L* is a function only of $\mathcal S$, and not alignment $\mathcal A$, so that all alternate alignments of $\mathcal S$ are normalized by the same quantity. Thus ranking alternate alignments by $F_\texttt {AC}$ orders them by the numerator: their predicted number of core columns.) The family of functions that we consider for the normalizer *L* of feature $F_\texttt {AC}$ are linear combinations of products of at most three factors from the following:

- aggregate measures of the lengths of sequences in $\mathcal S$, namely their minimum, mean, and maximum length;
- averages over all pairs of sequences in $\mathcal S$ of the ratio of their longest-common-subsequence length divided by an aggregate measure of the lengths of the pair of sequences (which can be viewed as forms of “percent identity”);
- averages over all pairs of sequences of the ratio of their difference in sequence length divided by an aggregate length measure (forms of “percent indel”); and
- averages over all pairs of sequences of the ratio of aggregate length measures for the pair (forms of “relative indel”).

More precisely, each term of the linear combination is a product whose factors are one aggregate length measure, and at most two average ratios from different groups in the above. Finally, we learn the normalizer from training data by solving a linear program to find coefficients of the linear combination that minimize its $L_1$-norm with the true number of core columns, across training protein benchmarks.

The final fitted function $L(\mathcal {S})$ that we use for the new Predicted Alignment Coreness feature is given later.

### Augmenting existing features by coreness

In addition to using the coreness regressor to directly estimate the accuracy of an alignment via the new feature function $F_\texttt {AC}$, we also augment some of the existing feature functions in Facet to concentrate their evaluation on columns with higher predicted coreness (since only on core columns is true accuracy measured). A full description of all feature functions in Facet is in [3]. The existing features that we augment using the coreness regressor are Secondary Structure Blockiness, Secondary Structure Identity, Amino Acid Identity, and Average Substitution Score. Each of these features can be viewed as a sum across columns of a quantity computed over all residue pairs in a column; in the augmented feature, this is now a *weighted* sum across columns, with columns weighted by their predicted coreness value. These augmented features are described in more detail below.

- *Secondary Structure Blockiness* $F_\texttt {BL}$ uses secondary structure predictions on the alignment’s proteins obtained from PSIPRED [14], and returns the maximum total score of an optimal packing of secondary structure blocks in the alignment, normalized by the total number of residue pairs in the alignment’s columns, where: a *block* is an interval of columns together with a subset of the sequences such that all residues in the block have the same secondary structure prediction, a *packing* is a set of blocks whose column intervals are all disjoint, and the *score* of a block is the total number of pairs of residues within the columns in the block. (So an optimal packing maximizes the number of pairs of residues in the alignment’s columns that are covered by blocks of consistent predicted secondary structure.) We create a new augmented feature $F'_\texttt {BL}$ by weighting the number of residue pairs for a column by the column’s predicted coreness value.
- *Secondary Structure Identity* $F_\texttt {SI}$ is the fraction of residue pairs in columns of the computed alignment that share the same predicted secondary structure. We create a new feature $F'_\texttt {SI}$ by weighting counts of column residue pairs by their column’s predicted coreness.
- *Amino Acid Identity* $F_\texttt {AI}$ is the fraction of column residue pairs that share the same amino-acid equivalence class. The augmented feature $F'_\texttt {AI}$ weights residue pairs by their column’s predicted coreness.
- *Average Substitution Score* $F_\texttt {AS}$ is the average BLOSUM62 score [20] of all column residue pairs, with BLOSUM similarity scores scaled to the range [0, 1]. The augmented feature $F'_\texttt {AS}$ weights this average by the column’s predicted coreness.

Other existing features not augmented by coreness that are used in their original form in the improved Facet estimator are the following. (Full details on these features are in [3].)

- *Secondary Structure Agreement* $F_\texttt {SA}$ uses predicted secondary structure confidences from PSIPRED (the confidence that a residue is in each of the three secondary structure states) to estimate the probability that each column residue pair shares the same secondary structure state, in a weighted window centered on each pair, and averages these estimates over all pairs.
- *Gap Open Density* $F_\texttt {GO}$ is the fraction of gap characters (‘-’) in the alignment that start a run of such characters.
- *Gap Extension Density* $F_\texttt {GE}$ is the fraction of alignment entries that are gap characters (‘-’).

The final improved Facet estimator that uses these features is given later.

## Assessing the coreness predictor

We evaluate our new approach to coreness prediction, and its use in accuracy estimation for alignment parameter advising, through experiments on a collection of protein multiple sequence alignment benchmarks. A full description of the benchmarks, and the universe of parameter choices for parameter advising, is given in [3].

Briefly, the benchmarks in our experiments consist of reference alignments of protein sequences largely induced by structurally aligning their known three-dimensional folded structures. We use the BENCH benchmark suite of Edgar [21], supplemented by a selection from the PALI benchmark suite of Balaji et al. [1]. Our full benchmark collection consists of 861 reference alignments.

We use 12-fold *cross-validation* to assess both column classification with our coreness predictor, and parameter advising with our augmented accuracy estimator. To correct for the overabundance of easy-to-align benchmarks when assessing parameter advising, we bin the benchmarks according to *difficulty*, measured by the true accuracy of their alignment computed by the Opal aligner [22, 23] under its default parameter setting. We ensure folds are balanced in their representation of benchmarks from all difficulty bins. For each fold, we generate a *training set* and *testing set* of example alignments by running Opal on each benchmark for each parameter choice from a fixed universe of 243 parameter settings.

### Constructing the coreness predictor

We first discuss results on learning the distance functions for the coreness predictor, and then discuss results on fitting its transform functions.

#### Learning the distance functions

To keep the size manageable of the linear program that we solve to learn the window class distance functions $d_c$, we use a training sample of 2000 total windows representing the structured classes. We find targets and impostors for windows from the training sample by performing nearest-neighbor searches in training sets that for each fold have 4000 windows from each structured window class. For each window from the training sample, the linear program uses 2 targets, and 150 impostors from each window class. For testing the accuracy of our learned coreness predictor, we use testing sets of 2000 total windows representing all classes (including the unstructured class). The windows for these training samples, training sets, and testing sets are drawn from corresponding training and testing example alignments.

We form the initial sets of targets and impostors for the linear program by either: (1) performing nearest-neighbor searches using a default distance function whose positional substitution score is a convex combination of (a) the VTML200 substitution score on the states’ amino acids (transformed to a dissimilarity value in the range [0, 1]), and (b) the identity function on the states’ secondary structure types, with positions weighted so the center column has twice the weight of its flanking columns; or (2) randomly sampling windows from the appropriate classes to choose targets and impostors, as further discussed below.

Once a distance function is learned by solving the linear program, we can *iterate* the process by using the learned distance function to recompute the sets of targets and impostors for another instance of the linear program, that is in turn solved to learn a new distance function. Table 1 shows results for this iterative process, where we use our coreness regressor to *classify* columns by simply thresholding the column’s predicted coreness value to obtain a binary classification of “core” (above the threshold) or “non-core” (at most the threshold). Beginning with the distance function learned at the first iteration from the initial default distance, Table 1 gives the *area under the curve* (AUC) measure for the receiver operating characteristic (ROC) curve, which implicitly considers all possible thresholds for the classifier, across ten iterations on both training and testing data.

**Table 1:** Core column classifier area-under-the-curve (AUC) for training and testing data with iterated distance learning

	Iteration									
	1	2	3	4	5	6	7	8	9	10
Training	88.7	94.3	99.2	99.4	99.5	99.7	99.6	99.7	99.4	99.7
Testing	84.7	81.3	80.8	80.0	83.4	82.5	81.2	80.9	80.3	81.6

Note that the training AUC steadily increases for the first four iterations, and then oscillates around a high plateau. This does not translate, however, into an improvement in the testing AUC, which actually drops and then oscillates at a much lower level.

While iterating distance learning markedly improves this core column classifier on the training examples, it is *overfitting*, and does not generalize well to testing examples. This may be due to the smaller training sample and training sets used to reduce the time for solving the linear program.

Interestingly, we found that using *random examples* from appropriate window classes for the target and impostor sets led to much better *generalization*. Specifically, this achieved a training and testing AUC of 86.0 and 88.6, respectively. Accordingly, in the remainder of the paper we use distance functions obtained by solving the linear program with random target and impostor sets, and no iteration, when assessing results on parameter advising.

#### Transforming distance to coreness

Figure 1 shows the fitted logistic functions $f_\text {core}$ and $f_\text {non}$ used to transform nearest-neighbor distance to predicted coreness, superimposed on the underlying true coreness data for one fold of training examples. The horizontal axis is nearest-neighbor distance $\delta$, while the vertical axis is the average true coreness of training examples at that distance (where this average is computed as detailed earlier). The blue and red curves show the average true coreness of training examples for which the nearest neighbor is in respectively either a core class or a structured non-core class. The top and bottom green curves show the logistic transform functions for respectively the core and non-core classes, fitted to this training data. Note that the green logistic curves fit the data quite well.

Interestingly, when a column’s window is sufficiently far away from all structured classes (including core and non-core classes), the green $f_\text {core}$ and $f_\text {non}$ logistic curves both converge to a predicted coreness around 33% (which roughly agrees with the blue and red empirical average coreness curves).

### Improving parameter advising

A *parameter advisor* and has two components: (1) an *accuracy estimator*, which estimates the accuracy of a computed alignment, and (2) an *advisor set*, which is a set of candidate assignments of values to the aligner’s parameters. The advisor picks the choice of parameter values from the advisor set for which the aligner yields the computed alignment of highest estimated accuracy.

In our parameter advising experiments, we assess the true accuracy of the multiple sequence alignment tool Opal [22, 23] combined with an advisor that uses the accuracy estimator Facet [3] (the best estimator for parameter advising in the literature), augmented by our new coreness predictor as well as by two other column-quality tools: TCS [11] and ZORRO [10]. We compare these advising results against prior approaches using for the estimator both the original unmodified Facet as well as TCS (the next-best estimator for parameter advising in the literature). We also compare against augmenting Facet by *true* coreness, which represents the unattainable limit reached by a perfect coreness predictor.

These experiments focus on advising for the Opal aligner, as it is an ideal test bed for studying parameter advising: in contrast to other aligners, at each node of the guide tree during progressive alignment, Opal computes subalignments that are *optimal* with respect to the given parameter choice for the sum-of-pairs scoring function with affine gap costs [24].

In parameter advising, the choice of advisor set is crucial, as the performance of the advisor is limited by the quality of the computed alignments generated by this set of parameter choices. We consider two types of advisor sets [25, 26]:

- estimator-independent *oracle sets*, which are learned for a conceptual oracle advisor that has access to true accuracy for its estimator, by solving an integer linear program to achieve optimal advising accuracy on training data; and
- estimator-aware *greedy sets*, which are learned for a specific concrete estimator by a greedy approximation algorithm that guarantees near-optimal training accuracy, and which tend to perform better than oracle sets in practice when used with their concrete estimator.

(We emphasize that when using oracle sets for the advisor set in our experiments, they are always used in conjunction with a concrete *imperfect* accuracy estimator.) These advisor sets are drawn from a larger *universe* of possible parameter choices. We use the universe of 243 parameter choices enumerated in [25].

**Fig. 2:**
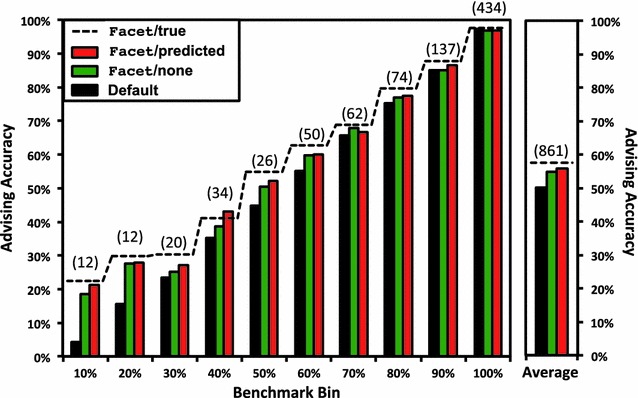
Advising accuracy within benchmark bins. This *bar chart* shows results within each bin of benchmarks, where bins group benchmarks by difficulty, for parameter advising with greedy advisor sets of cardinality 7. For each of the ten bins listed along the horizontal axis, the vertical axis gives advising accuracy, averaged over the benchmarks in the bin; on the *far right* is an average of all ten bin-averages. Bins are labeled on the horizontal axis by the upper limit of their difficulty range, where the difficulty of a benchmark is the true accuracy of its alignment computed by the Opal aligner under its default parameter setting. The *colored bars in each bin* show average advising accuracy for Opal using: its optimal default parameter setting, in *black*; advising with the original unaugmented Facet estimator, in *green*; and advising with Facet augmented by predicted coreness, in *red*. The *dashed line* shows average advising accuracy for each bin if Facet were augmented by true coreness: the limit achieved by a perfect coreness predictor. In *parentheses above the bars* is the number of benchmarks in each bin (while on the far right is their total number)

As mentioned earlier, we bin alignments according to difficulty to correct for the overabundance of easy-to-align benchmarks. Figure 2 lists in parentheses above the bars the number of benchmarks in each bin. When reporting advising accuracy, we give the true accuracy of the alignments chosen by the advisor, uniformly averaged over *bins* (rather than uniformly averaging over benchmarks). With this equal weighting of bins, an advisor that uses only the single optimal default parameter choice will achieve an average advising accuracy of roughly 50% (demonstrated by the black “default” bar on the far right in Fig. 2). This establishes, as a point of reference, advising accuracy 50% as the *baseline* against which to compare advising performance.

#### The augmented Facet estimator

We use our coreness predictor to modify the Facet accuracy estimator by including the new Predicted Alignment Coreness feature, and augmenting existing feature functions by coreness. We learned coefficients for these feature functions, as well as all the features originally in Facet, using the difference-fitting technique described in [3].

The new alignment accuracy estimator that uses our coreness predictor has non-zero coefficients for

- the new feature: Predicted Alignment Coreness $F_\texttt {AC}$ ;
- two features augmented by our coreness predictor: Secondary Structure Identity $F'_\texttt {SI}$ , and Secondary Structure Blockiness $F'_\texttt {BL}$ ; and
- five original unaugmented features: Secondary Structure Agreement $F_\texttt {SA}$ , Secondary Structure Identity $F_\texttt {SI}$ , Secondary Structure Blockiness $F_\texttt {BL}$ , Gap Extension Density $F_\texttt {GE}$ , and Gap Open Density $F_\texttt {GO}$ .

**Fig. 3:**
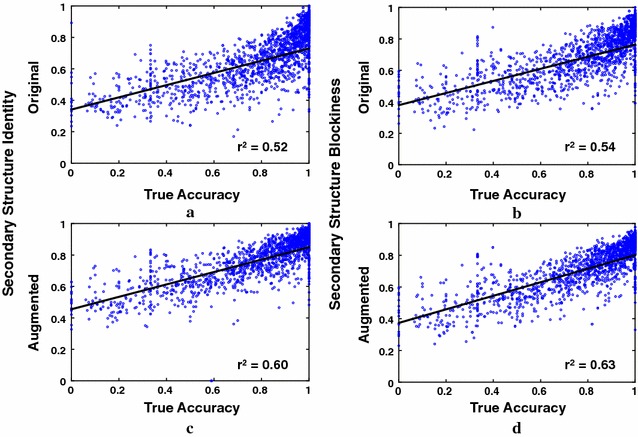
Correlation of augmented and unaugmented feature functions with true accuracy. The *scatterplots* show the correlation with true accuracy of alignment feature functions for the Facet accuracy estimator. Points in the scatterplots correspond to computed alignments for benchmarks with known reference alignments; all scatterplots are over the same set of alignments. The *vertical axis* is the feature function value, while the *horizontal axis* is true accuracy of the computed alignment with respect to the reference. The *top* and *bottom* scatterplots correspond respectively to unaugmented and augmented versions of the same feature function: on *top* is the original unaugmented Facet feature, and on the *bottom* is this feature augmented with predicted coreness. The plotted feature functions are: **a** original Secondary Structure Identity *F*_SI_, **b** original Secondary Structure Blockiness *F*_BL_, **c** augmented Secondary Structure Identity *F*′_SI_, and **d** augmented Secondary Structure Blockiness *F*′_BL_

To give an idea of how these augmented and unaugmented features behave, Fig. 3 shows the correlation between feature values and true accuracy for computed alignments. On the left is Secondary Structure Identity, on the right is Secondary Structure Blockiness, and on the top and bottom are respectively the *original* and *augmented* versions of these features. (For reference, least-squares lines are shown fitted to the data, where points are weighted so each accuracy decile has the same total weight.) Note in the scatterplots that the augmented features have somewhat *higher slope* and *lower spread* than their unaugmented versions. This can yield a stronger feature for discriminating high-accuracy from low-accuracy alignments, which may explain their inclusion in the new fitted estimator.

The resulting augmented accuracy estimator is

$$\begin{aligned} \begin{array}{l} (0.656) \, F'_\texttt {SI} + (0.128) \, F'_\texttt {BL} + (0.123) \, F_\texttt {SA} + (0.089) \, F_\texttt {SI} \,+ (0.064) \, F_\texttt {BL} \,+ \\ (0.015) \, F_\texttt {AC} + (0.007) \, F_\texttt {GE} + (0.006) \, F_\texttt {GO}.\end{array} \end{aligned}$$

(The above coefficients are fitted over *all* benchmarks; in our cross-validation experiments, the estimator used for each fold is fitted only over the benchmarks in the *training* set for that fold.) We mention that these feature functions have different ranges, so the magnitudes of their coefficients should not necessarily be interpreted in terms of the importance of the feature.

**Fig. 4:**
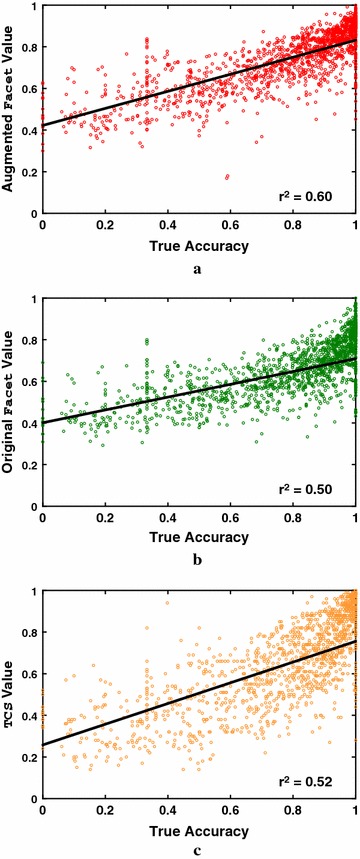
Correlation of estimators with true accuracy. The *scatterplots* show the correlation with true accuracy of alignment accuracy estimators. Points correspond to computed alignments for benchmarks; all *scatterplots* show the same alignments. The *vertical axis* is the estimator value; the *horizontal axis* is alignment true accuracy. The plotted accuracy estimators are: **a** the Facet estimator augmented with predicted coreness, **b** the original unaugmented Facet estimator, and **c** the TCS estimator

To illuminate how the new augmented estimator behaves, Fig. 4 shows the correlation on computed alignments between estimator value and true accuracy for the final augmented Facet estimator, original unaugmented Facet, and the TCS estimator. (Fitted least-squares lines are shown, with points weighted so each accuracy decile has the same weight.) Note that augmented Facet again has somewhat *higher slope* and *lower spread* than its original version. On the other hand, TCS has the highest slope, but also the highest spread. This may explain why augmented Facet performs better for parameter advising (as shown later in our experiments).

#### The coreness feature normalizer

The normalizer function $L(\mathcal {S})$ used in Predicted Alignment Coreness (feaure $F_\texttt {AC}$), gives an estimate of the number of core columns in the unknown reference alignment of sequences $\mathcal S$, and is a linear combination of basic measures of $\mathcal S$, as described earlier. Optimal coefficients for this linear combination are learned by minimizing its $L_1$-norm with the true number of core columns in training benchmarks.

The fitted estimator $L(\mathcal {S})$ for Predicted Alignment Coreness is

$$\begin{aligned} \begin{array}{l@{}l} (1.020)  \ell _\text {min} \, p_\text {max} \, q_\text {min} &{} + (0.151)  \ell _\text {min} \, q_\text {min} \,+ (0.035) \,\ell _\text {avg} \, p_\text {max} \, q_\text {avg} \,+\, \\ (0.032)  \ell _\text {avg} \, p_\text {min} \, r_\text {min} &{} \,+\, (0.003)  \ell _\text {max} \, p_\text {avg} \, r_\text {avg}, \end{array} \end{aligned}$$

where

- $\ell _\text {min}, \, \ell _\text {avg}, \, \ell _\text {max}$ are respectively the minimum, average, and maximum sequence lengths in $\mathcal S$;
- $p_\text {min}, \, p_\text {avg}, \, p_\text {max}$, similar to percent identity measures, are the longest-common-subsequence length for each pair of sequences normalized by respectively the minimum, average, and maximum sequence length for the pair, averaged over all pairs;
- $q_\text {min}, \, q_\text {avg}$ are quotients of respectively the minimum or average sequence length for each pair of sequences divided by the maximum length for the pair, averaged over all pairs; and
- $r_\text {min}, \, r_\text {avg}$ are ratios of the difference in sequence lengths for each pair of sequences divided by respectively the minimum and average sequence length for the pair, averaged over all pairs.

(The above coefficients are fitted over *all* benchmarks; in our cross-validation experiments, the normalizer for each fold is fitted only over the *training* benchmarks for that fold.)

**Fig. 5:**
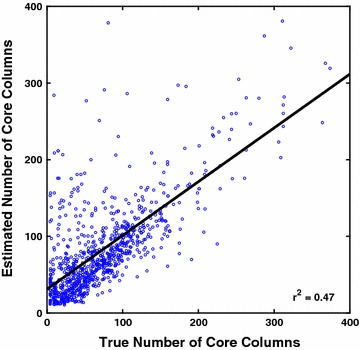
Correlation of the estimated and true number of core columns. Each point in the scatterplot corresponds to a reference alignment from the collection of 861 benchmarks. The *horizontal axis* is the true number of core columns in the alignment, while the *vertical axis* is the estimated number of core columns for the alignment’s sequences $\mathcal S$, computed using the fitted function $L(\mathcal {S})$ given earlier when discussing the coreness feature normalizer

Figure 5 shows in a scatterplot the correlation between this *estimate* of the number of core columns and the *true* number of core columns for each benchmark. While the fitted estimator does correlate with the true number of core columns, it tends to overestimate, possibly due to larger benchmarks having more columns that are close to—but not quite—core.

#### Performance on parameter advising

We assess the performance of parameter advising when the advisor uses for its accuracy estimator:

- the augmented Facet estimator (“Facet/predicted”),

in comparison with

- the original unaugmented Facet estimator (“Facet/none”),
- Facet augmented by TCS (“Facet/TCS”),
- Facet augmented by ZORRO (“Facet/ZORRO”), and
- Facet augmented by true coreness (“Facet/true”).

We also compare with

- TCS (the next-best estimator for advising in the literature).

For the advisor set of parameter choices that the advisor picks from using these estimators, we consider both oracle and greedy sets [26].

#### Performance when expanding the parameter choices

**Fig. 6:**
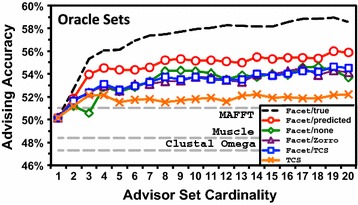
Advising accuracy using oracle sets. This figure plots average advising accuracy using oracle advisor sets with different estimators, at varying set cardinalities. The *horizontal axis* is the cardinality of the advisor set: the number of parameter choices from which the advisor selects. The *vertical axis* is average true accuracy of the parameter advisor, where the average accuracy within each difficulty bin is then averaged across bins. The *curves* plot advisors using: the Facet estimator augmented by predicted coreness, the original Facet estimator with no augmentation, Facet augmented by TCS column quality scores, Facet augmented by ZORRO quality scores, and using TCS as the estimator. The *dashed black curve* is Facet augmented by true coreness: the limit attained with a perfect coreness predictor. As baselines for comparison, the *dashed grey lines* are the average accuracies of the standard aligners MAFFT, MUSCLE, and Clustal Omega, under their default parameter settings

**Fig. 7:**
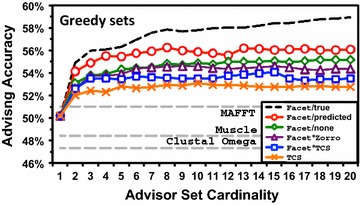
Advising accuracy using greedy sets. Similar to Fig. 6, this plots average advising accuracy using greedy advisor sets learned for different estimators. The *horizontal axis* is the cardinality of the advisor set; the *vertical axis* is the average true accuracy of the parameter advisor. The *curves* plot the average accuracy of advisors that use greedy sets learned for the following estimators: Facet augmented by predicted coreness, unaugmented Facet, Facet augmented by TCS, Facet augmented by ZORRO, and TCS alone. The *dashed black curve* represents Facet augmented by true coreness. The *dashed grey lines* are the average accuracies of MAFFT, MUSCLE and Clustal Omega using default parameter settings

Figures 6 and 7 show parameter advising performance using oracle and greedy advisor sets, respectively. In both figures, the horizontal axis is advisor set cardinality (the number of different parameter choices available to the advisor), while the vertical axis is advising accuracy for testing folds (the true accuracy on testing benchmarks of the aligner combined with the parameter advisor), uniformly averaged across bins. The curves show performance with the Opal aligner [22, 23]. For reference, the default alignment accuracy for three other popular aligners, MAFFT [27], MUSCLE [28], and Clustal Omega [29], is also shown with dashed horizontal lines.

Figure 6 shows that on *oracle* advisor sets, Facet/predicted compared to Facet/none boosts the average accuracy of parameter advising by more than 3%. This increase is in addition to the improvement of Facet over TCS.

Figure 7 shows that on *greedy* advisor sets, Facet/predicted boosts advising accuracy as well: for example, at cardinality 7, by more than 1%. (Note that accuracies for the greedy set curves are already higher than for oracle sets.) Up to cardinality 7, the accuracy for Facet/predicted is about halfway between Facet/none and Facet/true (the unattainable perfect coreness predictor). Interestingly, Facet/TCS and Facet/ZORRO actually have worse accuracy than Facet/none.

#### Performance when generalizing to new data

**Fig. 8:**
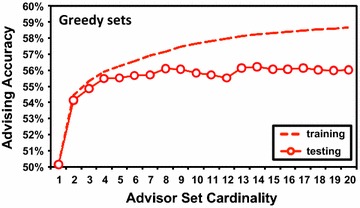
Training and testing accuracy using greedy advisor sets. The figure plots average advising accuracy on training and testing benchmarks, using greedy advisor sets learned for the Facet accuracy estimator augmented by predicted coreness. The *horizontal axis* is advisor set cardinality; the *vertical axis* is advising accuracy uniformly averaged across difficulty bins. The *dashed* and *solid curves* give accuracies for training and testing benchmarks respectively, averaged over cross-validation folds. Since each benchmark is in the testing set of exactly onefold, and the training set of all other folds, averaging over folds uniformly averages over benchmarks

While with *greedy* advisor sets, using predicted coreness to augment Facet does boost advising accuracy, a larger improvement might be realized by pairing with a better approach to finding estimator-aware advisor sets than greedy set-finding. The boost in accuracy we observe may actually be limited by the methods we are using to find advisor sets for the improved estimator. As an indication, Fig. 8 contrasts average *training* and *testing* accuracy for advising with Facet/predicted on greedy sets: specifically, the accuracy using greedy sets that were learned on training benchmarks when these same sets are applied to testing benchmarks. The upper dashed curve is average training advising accuracy, while the lower solid curve is testing accuracy. The drop between these curves indicates greedy set-finding is *overfitting* to training data, and not generalizing well to testing data. With better generalization, we might also continue to get improved performance at set sizes beyond cardinality 7, where greedy advisor sets currently plateau.

#### Performance within difficulty bins

Advising accuracy within difficulty *bins* for greedy sets of cardinality 7 is shown earlier in Fig. 2. In this bar chart, for the bin at each difficulty on the horizontal axis, advising accuracy averaged over just the benchmarks in the bin is shown on the vertical axis. The final chart on the right gives accuracy averaged across all bins. On difficult benchmarks, Facet/predicted boosts the accuracy of Facet/none by more than 3%. Note also how close Facet/predicted is to Facet/true: the advising accuracy is already quite close to what could be achieved augmenting with a *perfect* coreness predictor.

For a point of reference, advising accuracy uniformly-averaged over *benchmarks* (rather than bins), on greedy sets of cardinality 10, is: for Facet/none, 81.9%; and for Facet/predicted, 82.2%. By comparison, on these same benchmarks the corresponding average accuracy of other popular aligners using their default parameter settings is: Clustal Omega, 77.3%; MUSCLE, 78.1%; MAFFT, 79.4%; and Opal, 80.5%.

## Conclusion

We have developed a column coreness predictor for protein multiple sequence alignments that uses a regression function on nearest neighbor distances for class distance functions learned by solving a new linear programming formulation. When applied to alignment accuracy estimation and parameter advising, the coreness predictor strongly outperforms other column confidence estimators from the literature, and provides a substantial boost in advising accuracy.

### Further research

A key issue left to explore is how to improve the *generalization* of distance-function learning and greedy advisor-set learning. Currently both tend to overfit to training data, resulting in a loss of testing accuracy. One way to address overfitting in distance learning would be to lower the number of substitution-score parameters in the learned distance functions by using reduced protein-sequence alphabets with amino-acid equivalence classes, which should aid generalization.

Another very promising research direction is to apply the improved accuracy estimator to *ensemble multiple sequence alignment* [5], where the estimator is used to pick the alignment output by an ensemble of sequence aligners. Any improvement in the estimator should yield further accuracy boosts for ensemble alignment.
